# Ultrasound- and Thermo-Responsive Ionic Liquid Polymers

**DOI:** 10.3390/polym10030301

**Published:** 2018-03-11

**Authors:** Kohei Itsuki, Yuuki Kawata, Komol Kanta Sharker, Shin-ichi Yusa

**Affiliations:** Department of Applied Chemistry, Graduate School of Engineering, University of Hyogo, 2167 Shosha, Himeji, Hyogo 671-2280, Japan; flowing.yumeniji.quiz@gmail.com (K.I.); yuki.chibikitty.1020@gmail.com (Y.K.); sharkerkomol@diu.edu.bd (K.K.S.)

**Keywords:** thermo-responsive, ultrasound, reversible addition-fragmentation chain transfer (RAFT) polymerization, ionic liquid polymer, electrostatic repulsion, hydrophobic interaction, lower critical solution temperature (LCST)

## Abstract

Poly(sodium 2-acrylamido-2-methylpropanesulfonate) (PAMPSNa) was prepared via reversible addition-fragmentation chain transfer (RAFT) radical polymerization. An ionic liquid polymer (PAMPSP_4448_) was then prepared by exchanging the pendant counter cation from sodium (Na^+^) to tributyl-*n*-octylphosphonium (P_4448_^+^). We studied the ultrasound- and thermo-responsive behaviors of PAMPSP_4448_ in water. When the aqueous PAMPSP_4448_ solution was heated from 5 to 50 °C, the solution was always transparent with 100% transmittance. Unimers and interpolymer aggregates coexisted in water in the temperature range 5–50 °C. Generally, hydrogen bonding interactions are broken as the temperature increases due to increased molecular motion. Above 25 °C, the size of the interpolymer aggregates decreased, because hydrophobic interactions inside them were strengthened by dehydration accompanying cleavage of hydrogen bonds between water molecules and the pendant amide or sulfonate groups in PAMPSP_4448_. Above 25 °C, sonication of the aqueous solution induced an increase in the collision frequency of the aggregates. This promoted hydrophobic interactions between the aggregates to form larger aggregates, and the aqueous solution became turbid. When the temperature was decreased below 8 °C, hydrogen bonds reformed between water molecules and the pendant amide or sulfonate groups, allowing PAMPSP_4448_ to redissolve in water to form a transparent solution. The solution could be repeatedly controlled between turbidity and transparency by sonication and cooling, respectively.

## 1. Introduction

The chemical and physical properties of stimuli-responsive polymers can be controlled by external stimuli such as temperature, pH, and light. Various studies on stimuli-responsive polymers have been reported, concerning their application to drug delivery systems and surface coatings for cell culture dishes [1,2,3,4,5]. Thermo-responsive polymers have been widely used as stimuli-responsive polymers. Their lower critical solution temperature (LCST) behavior in water is particularly well known [6,7]. These polymers can dissolve in water below the LCST; however, they cannot dissolve above the LCST. Recently, Kohno et al. [8,9] reported that LCST behavior was observed at 57 °C for aqueous poly(tetrabutylphosphonium styrenesulfonate) (PSSP_4444_), which was prepared by exchanging the counter cation, sodium (Na^+^) in poly(sodium styrenesulfonate) (PSSNa), with tetrabutylphosphonium (P_4444_^+^). The monomer, tetrabutylphosphonium styrenesulfonate in PSSP_4444_, is an ionic liquid. PSSP_4444_ dissolves in water via hydrogen bonding interactions between water molecules as donors and oxygen atoms in the pendant sulfonate groups as acceptors below the LCST [10]. However, above the LCST, dehydration accompanies destruction of the hydrogen bonds, and the hydrophobic polymer chains then aggregate and are precipitated. An ionic liquid polymer, poly(tributyl-4-vinylbenzyl phosphonium hexylsulfonate), which was prepared by exchanging the counter chloride anion in poly(tributyl-4-vinylbenzyl phosphonium chloride) to the hexylsulfonate anion, shows LCST-type thermo-responsive behavior at 53 °C in water [11]. In this manner, conventional polyelectrolytes can sometimes be used to synthesize ionic liquid polymers by exchanging the counter ion to endow thermo-responsive behavior.

Sonication is often used to increase molecular motion to break and disperse non-covalent aggregates [12,13]. Ultrasound is also used to achieve decomposition by breaking chemical bonds. Some research groups reported that ultrasound can be used as an external stimulus to change the association state. Diblock copolymers composed of a hydrophilic poly(ethylene oxide) (PEO) block and a hydrophobic poly(2-tetrahydropyranyl methacrylate) (PTHPMA) block can form polymer micelles in water with hydrophobic PTHPMA cores and hydrophilic PEO shells [14,15]. When the aqueous polymer solution was sonicated, the PTHPMA block changed to hydrophilic poly(methacrylic acid) due to hydrolysis of its pendant ester groups. The hydrophobic core then became hydrophilic to dissociate the micelle. Although sonication is mainly used to increase dispersibility and induce cleavage of weak chemical bonds by providing high energy to molecules, ultrasound induced association phenomena were reported. Naota et al. [16,17] reported that sonication of binuclear Pt complexes in an organic solvent formed a gel due to intermolecular interactions, and the gel was returned to a sol state by heating.

**Figure 1:**
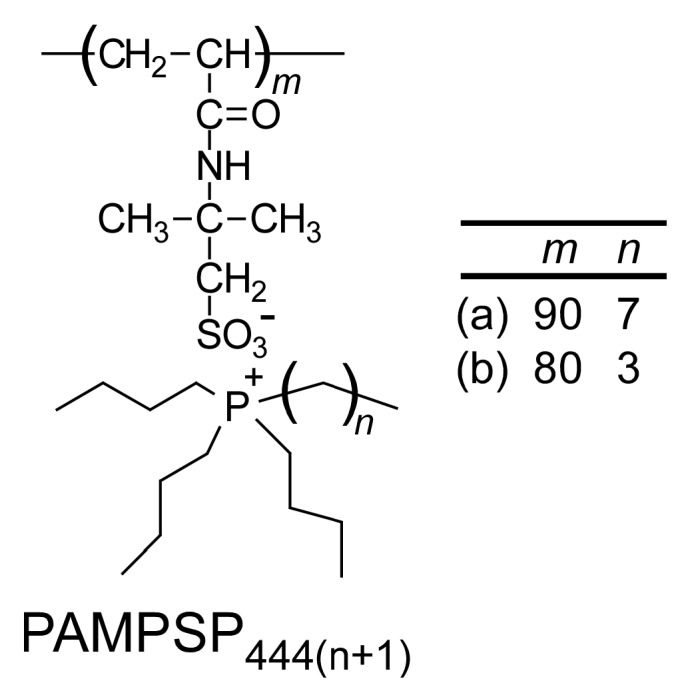
Chemical structures of (**a**) PAMPSP_4448_ and (**b**) PAMPSP_4444_.

In this study, poly(sodium 2-acrylamido-2-methylpropanesulfonate) (PAMPSNa) was prepared via RAFT radical polymerization, and the counter cation, Na^+^, was then exchanged with tributyl-*n*-octylphosphonium (P_4448_^+^) or tetrabutylphosphonium (P_4444_^+^) cations to obtain two ionic liquid polymers (PAMPSP_4448_ and PAMPSP_4444_) (Figure 1). The aqueous PAMPSP_4444_ solution did not show ultrasound- or thermo-responsive behavior. However, the aqueous PAMPSP_4448_ solution could be repeatedly controlled between turbidity and transparency via sonication and cooling, respectively. We studied the ultrasound- and thermo-responsive behavior of the aqueous PAMPSP_4448_ solution using percent transmittance (%*T*), light scattering, transmission electron microscopy (TEM), and fluorescence probe techniques.

## 2. Materials and Methods

### 2.1. Materials

2-Acrylamido-2-methylpropanesulfonic acid (AMPS, 98%, Tokyo Chemical Industry, Tokyo, Japan), 4,4′-azobis(4-cyanovaleric acid) (V-501, 98%, Wako Pure Chemical, Osaka, Japan), tetrabutylphosphonium bromide (P_4444_Br, 95%, Wako Pure Chemical), and tributyl-*n*-octylphosphonium bromide (P_4448_Br, 98%, Tokyo Chemical Industry) were used as received. 4-Cyanopentanoic acid dithiobenzoate (CPD) was synthesized as previously reported [18]. Pyrene (97%, Wako Pure Chemical) was purified by recrystallization from ethanol. Methanol was dried with 3A molecular sieves and purified by distillation. Water was purified with an ion exchange column system. Other reagents were used as received.

### 2.2. Synthesis of Poly(Sodium 2-Acrylamido-2-Methylpropanesulfonate) (PAMPSNa)

A typical procedure for preparing poly(sodium 2-acrylamido-2-methylpropanesulfonate) (PAMPSNa) is as follows [19]. AMPS (5.00 g, 24.1 mmol) was neutralized with NaOH to adjust it to pH 6.0 in water (150 mL). CPD (67.4 mg, 0.24 mmol) and V-501 (27.1 mg, 0.097 mmol) were added to the solution. This was heated at 70 °C for 4 h under argon. The conversion of AMPS was estimated from ^1^H NMR (= 91.0%). After polymerization, the solution was dialyzed against pure water for two days using a dialysis membrane (MWCO = 14,000). The polymer (PAMPSNa) was recovered by freeze-drying (4.85 g, 86.7%). The number-average molecular weight (*M*_n_(NMR)) and degree of polymerization (DP(NMR)) were 2.09 × 10^4^ and 90, respectively, estimated from ^1^H NMR. The molecular weight distribution (*M*_w_/*M*_n_) was 1.18, estimated from gel-permeation chromatography (GPC).

### 2.3. Synthesis of Poly(Tributyl-N-Octylphosphonium 2-Acrylamido-2-Methylpropanesulfonate) (PAMPSP_4448_)

PAMPSNa (0.500 g, 0.0265 mmol, *M*_n_(NMR) = 2.09 × 10^4^, *M*_w_/*M*_n_ = 1.18) and P_4448_Br (8.63 g, 21.8 mmol) were dissolved in methanol (21.8 mL). The solution was heated at 60 °C for 48 h. After the reaction, the mixture was dialyzed against pure water for three days. The polymer (PAMPSP_4448_) was recovered by freeze-drying (0.617 g, 49.8%). *M*_n_(NMR) and *M*_w_/*M*_n_ were 4.68 × 10^4^ and 1.22, respectively.

### 2.4. Synthesis of Poly(Tetrabutylphosphonium 2-Acrylamido-2-Methylpropanesulfonate) (PAMPSP_4444_)

PAMPSP_4444_ was prepared as a reference sample. PAMPSNa (1.00 g, 0.0467 mmol, *M*_n_(NMR) = 2.14 × 10^4^, *M*_w_/*M*_n_ = 1.39) and P_4444_Br (14.8 g, 43.5 mmol) were dissolved in methanol (87.5 mL). The solution was heated at 60 °C for 48 h. After the reaction, the mixture was dialyzed against pure water for three days. The polymer (PAMPSP_4448_) was recovered by freeze-drying (1.12 g, 65.8%). *M*_n_(NMR) and *M*_w_/*M*_n_ were 3.75 × 10^4^ and 1.29, respectively.

### 2.5. Preparation of Aqueous Polymer Solution

Predetermined amounts of PAMPSP_4448_ and PAMPSP_4444_ were dissolved in water. The aqueous solution was cooled in an ice bath for 5 min at 0 °C. Sonication was performed with a 2510J-MT apparatus (125 W, 42 kHz, Branson, Atsugi, Japan).

### 2.6. Measurements

^1^H NMR spectra were measured with a DRX-500 NMR spectrometer (Bruker, Yokohama, Japan) operating at 500 MHz. GPC measurements of PAMPSNa were performed with a DP8020 pump (Tosoh, Tokyo, Japan) and an RI8020 refractive index detector (Tosoh, Tokyo, Japan). An Asahipak SB-804 HQ column (Shodex, Tokyo, Japan) was used. A mixed solvent of phosphate buffer at pH 8.0 and acetonitrile (9/1, *v*/*v*) was used as the eluent at a flow rate of 0.6 mL/min at 40 °C. *M*_n_(GPC) and *M*_w_/*M*_n_ were calibrated using PSSNa standards. GPC measurements of PAMPSP_4448_ and PAMPSP_4444_ were performed with a PU-2085 Plus pump (Jasco, Tokyo, Japan) and an RI SE-61 refractive index detector (Shodex, Tokyo, Japan). A 7.0 μm bead size GF-7M HQ column (Shodex, Tokyo, Japan) was used. Methanol containing LiClO_4_ (0.1 M) was used as the eluent at a flow rate of 0.6 mL/min at 40 °C. *M*_n_(GPC) and *M*_w_/*M*_n_ for the samples were calibrated using PEO standards. Percent transmittance (%*T*) measurements were performed using a V-630 BIO UV-vis spectrometer (Jasco, Tokyo, Japan) with a 10 mm path length quartz cell. The temperature was controlled using a Jasco ETC-717 thermostat system (Jasco, Tokyo, Japan). The %*T* at 800 nm for the aqueous solution was monitored while heating from 0 to 50 °C, and while cooling from 50 to 0 °C at 1 °C/min with stirring. Phase-contrast microscopic observation was performed using a BZ-8000 fluorescence microscope (Keyence, Osaka, Japan) with a Plan Fluor ELWD DM 20× NAO 0.45 objective lens (Nikon, Tokyo, Japan). Dynamic light scattering (DLS) measurements were performed using a Zetasizer Nano-ZS instrument (Malvern, Worcestershire, UK) equipped with an He–Ne laser (4 mW). The obtained data were analyzed using Zetasizer Software 7.11 (Malvern, Worcestershire, UK). Transmission electron microscopy (TEM) was performed using a TEM-2100 microscope (JEOL, Tokyo, Japan) operated at an accelerating voltage of 200 kV. TEM samples were prepared by placing one drop of aqueous PAMPSP_4448_ solution on a copper grid coated with thin films of Formvar. Excess water was removed using a filter paper. The samples were stained with sodium phosphotungstate and dried under vacuum. Fluorescence emission measurements were performed using an F-2500 fluorescence spectrophotometer (Hitachi, Tokyo, Japan). Pyrene was used as a hydrophobic fluorescence probe. The polymer concentration was maintained at 0.5 g/L. PAMPSP_4448_ was dissolved in saturated aqueous pyrene solution (2.07 × 10^−7^ M). Fluorescence emission spectra were obtained at 25 °C with an excitation wavelength of 334 nm, and the excitation and emission slit widths were maintained at 20 and 2.5 nm, respectively.

## 3. Results and Discussion

### 3.1. Synthesis of Polymers

Ionic liquid polymer, PAMPSP_4448_, was synthesized by exchanging the pendant counter cation from Na^+^ to P_4448_^+^ in PAMPSNa prepared via RAFT radical polymerization (Figure S1). The reference polymer, PAMPSP_4444_, was also synthesized using the same method. The percent conversion (*p*) was estimated from decreases in the ^1^H NMR peak area integrated intensity ratios of vinyl protons at 5.6, 6.1, and 6.2 ppm in AMPS monomer during the polymerization. The theoretical number-average molecular weight (*M*_n_(theory)) was calculated using the following equation:

(1) $$M_{n}\left(\mathrm{theory}\right)=\frac{{\left[M\right]}_{0}}{{\left[\mathrm{CTA}\right]}_{0}}\times \frac{p}{100}\times M_{m}+M_{\mathrm{CTA}}$$

where [M]_0_ is the initial molar concentration of the monomer, [CTA]_0_ is the initial molar concentration of CTA, *M*_m_ is the molecular weight of the monomer, and *M*_CTA_ is the molecular weight of CTA. The *M*_n_ and *M*_w_/*M*_n_ values of the obtained polymers are summarized in Table 1.

**Figure 2:**
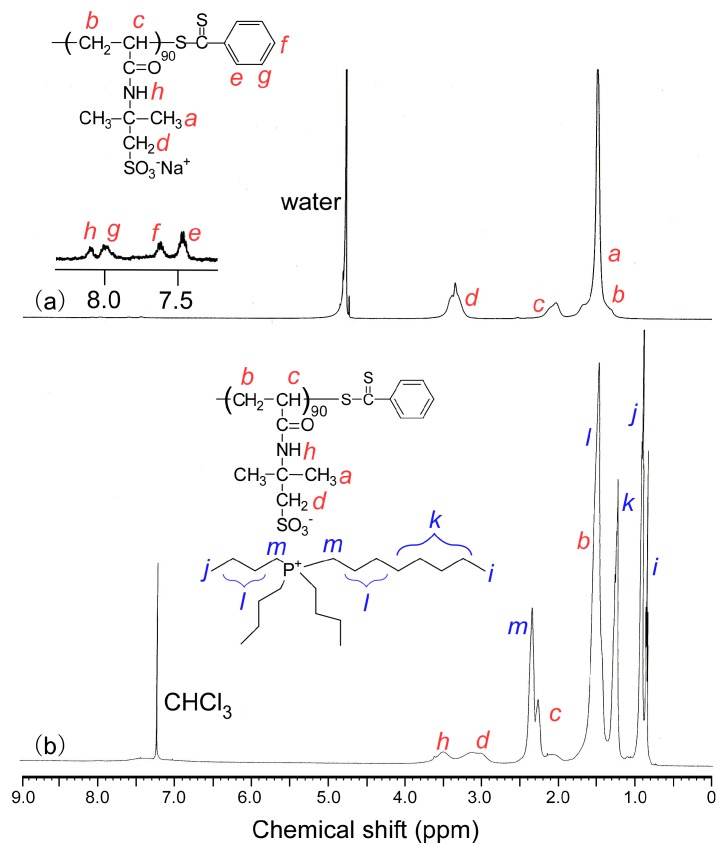
^1^H NMR spectra for (**a**) PAMPSNa in D_2_O and (**b**) PAMPSP_4448_ in CDCl_3_.

The DP(NMR) and *M*_n_(NMR) of PAMPSNa were calculated as 90 and 2.09 × 10^4^, respectively, by comparing the ^1^H NMR integrated intensity ratio of the pendant methylene protons at 3.2 ppm to the terminal phenyl protons at 7.4–8.1 ppm in D_2_O (Figure 2a). The obtained PAMPSNa had a well-controlled structure, because *M*_n_(theory) and *M*_n_(NMR) were close, and *M*_w_/*M*_n_ was narrow.

Methylene protons attributed to the octyl group at 1.2 ppm and methylene protons adjacent to the phosphorous atom in P_4448_ at 2.3 ppm could be observed in the ^1^H NMR spectrum for PAMPSP_4448_ in CDCl_3_ (Figure 2b). The exchange ratio (ER) of Na^+^ to P_4448_^+^ was 95.6%, estimated from the integrated area intensity ratio of the pendant methylene protons in PAMPS at 2.8–3.3 ppm to the methyl protons in P_4448_ at 0.8–0.9 ppm. The phenyl protons at the polymer chain end could be observed for PAMPSNa at 7.4–8.1 ppm but not for PAMPSP_4448_. During the exchange reaction involving the counter cation, most of the terminal dithiobenzoate groups may have been decomposed. Similarly, the ER for the reference polymer, PAMPSP_4444_, was 99.7% (Figure S2).

### 3.2. Turbidimetry

**Figure 3:**
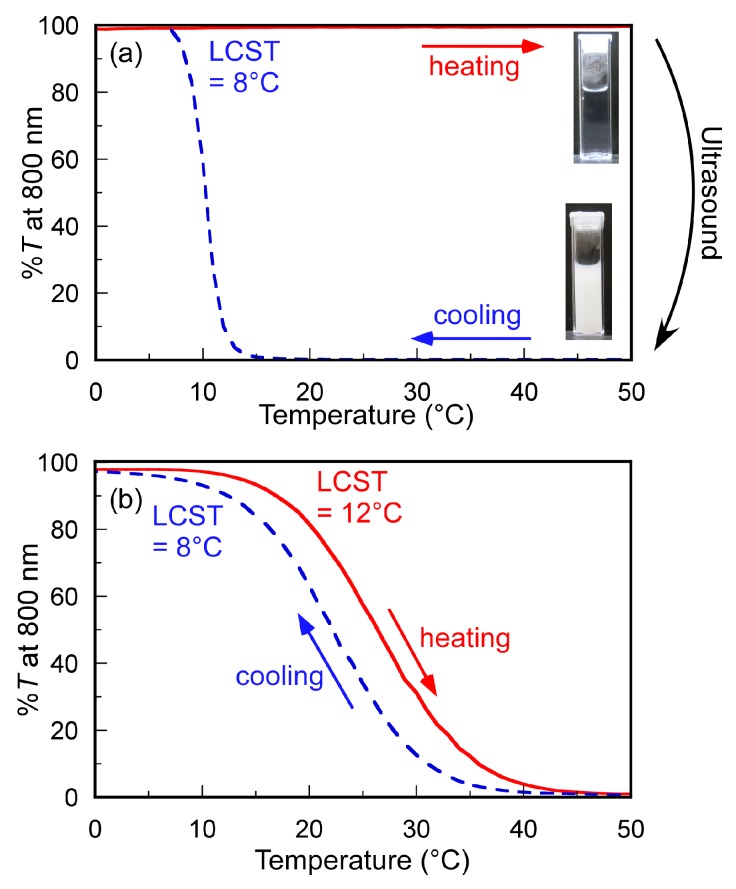
Percent transmittance (%*T*) at 800 nm for PAMPSP_4448_ in (**a**) pure water and (**b**) 0.1 M aqueous NaCl as a function of temperature at *C*_p_ = 5 g/L. The temperature was increased from 0 to 50 °C (heating; —) and decreased from 50 to 0 °C (cooling, ---) at 1 °C/min with stirring. PAMPSP_4448_ in pure water was sonicated for 10 min at 50 °C and then cooled.

When the temperature of the aqueous PAMPSP_4448_ solution with *C*_p_ = 5.0 g/L was changed from 5 to 50 °C and from 50 to 5 °C at 1 °C/min, %*T* was constant at 100%, regardless of the temperature (Figure 3a). However, when the aqueous PAMPSP_4448_ solution was heated to 50 °C and sonicated at 50 °C for 10 min, it became turbid with a decrease in %*T* (Video S1). We investigated the relationship between the sonication time and the %*T* of the solution (Figure S3). A decrease in %*T* was observed when the sonication time was increased up to 3 min. When sonication was performed for more than 4 min, %*T* decreased to 0%. While the %*T* of the aqueous PAMPSP_4448_ solutions with *C*_p_ = 0.5 and 1.0 g/L remained constant (= 100%) regardless of the temperature, the solution became turbid upon sonication at 50 °C for 10 min (Figure S4). Hydrophobic interactions of the pendant alkyl chains in P_4448_ may induce the formation of large aggregates, presumably because of an increase in the collision frequency of interpolymer chains, causing the solution to become turbid. This phase separation behavior upon sonication will be discussed in more detail later in conjunction with the results of light scattering, TEM, and fluorescence probe techniques. When the sonicated turbid aqueous solution with *C*_p_ = 5.0 g/L was cooled below 8 °C, %*T* returned to 100% (Figure 3a). With *C*_p_ = 0.5 and 1.0 g/L, %*T* returned to 100% below 10 °C (Figure S4). The sonicated turbid solution became clear with cooling, probably because the large aggregates were hydrated and dissociated, allowing them to dissolve in water. As the temperature decreased, the molecular motion decreased to allow hydrogen bonds to form between water molecules and the pendant amide or sulfonate groups in PAMPSP_4448_.

The %*T* of the unsonicated aqueous PAMPSP_4448_ solution was almost 100% regardless of temperature. This observation suggested that further interpolymer aggregation due to hydrophobic interactions between alkyl chains in P_4448_ may be suppressed by electrostatic repulsion of the pendant sulfonate groups. Electrostatic repulsion of polyelectrolytes can be screened by adding low-molecular-weight salts such as NaCl [20]. We measured the temperature dependence of the %*T* of the aqueous PAMPSP_4448_ solution containing 0.1 M NaCl (Figure 3b). A decrease in %*T* was observed as temperature increased from 12 °C. As the temperature decreased from 50 °C, %*T* increased and reached 100% at temperatures below 8 °C. The hydrophobic interactions of the alkyl chains in P_4448_ became stronger in aqueous NaCl, because the electrostatic repulsion of the sulfonate anions in PAMPSP_4448_ was screened. Therefore, the aqueous NaCl showed LCST behavior. This observation suggested that heat-induced interpolymer aggregation was suppressed in pure water, because the electrostatic repulsion of the pendant sulfonate groups outweighs the hydrophobic interactions of alkyl chains in P_4448_.

The influence of the chemical structure of the counter cation on ultrasound- and thermo-responsive behaviors was investigated. We studied the temperature dependence of the %*T* of aqueous PAMPSP_4444_ solution containing P_4444_ with four butyl groups as a counter cation (Figure S5). As aqueous PAMPSP_4444_ solutions with and without NaCl at *C*_p_ = 5.0 g/L were heated to 50 °C, no changes in %*T* were observed. Though the solutions were sonicated at 50 °C for 10 min, %*T* remained almost constant at 100%. The aqueous PAMPSP_4444_ solution does not exhibit ultrasound- or thermo-responsive behaviors. The alkyl chain length of the counter cation is thus important with respect to ultrasound- and thermo-responsive behaviors.

**Figure 4:**
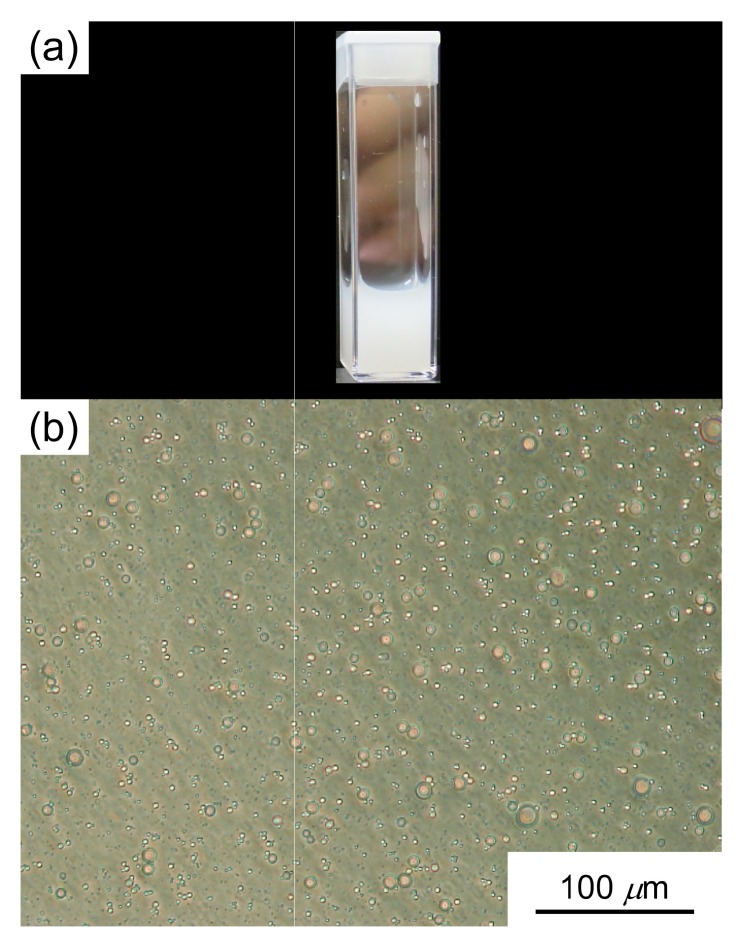
(**a**) Digital photograph and (**b**) phase-contrast microscope images of the turbid aqueous PAMPSP_4448_ solution at *C*_p_ = 5 g/L after sonication.

Sonication of the aqueous PAMPSP_4448_ solution at 50 °C induced turbidity. The turbid aqueous solution was observed with a phase-contrast microscope (Figure 4). Spherical large aggregates with an average radius of 2.5 μm could be observed. The sonicated turbid aqueous PAMPSP_4448_ solution cannot be measured by DLS due to its high turbidity.

### 3.3. Light Scattering

**Figure 5:**
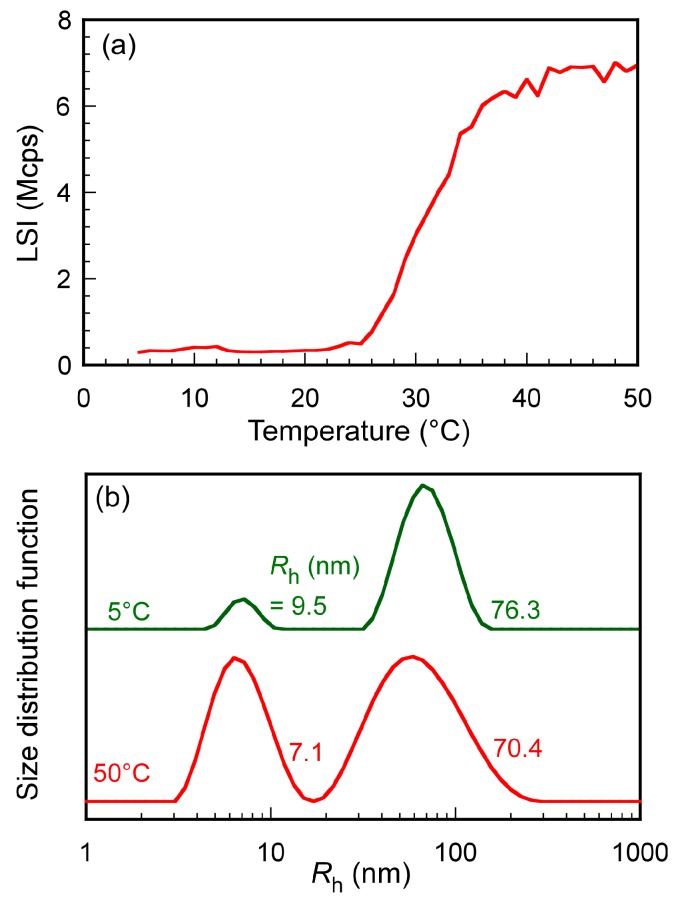
(**a**) Light scattering intensity (LSI) for aqueous PAMPSP_4448_ solution as a function of temperature. (**b**) Hydrodynamic radius (*R*_h_) distributions for unsonicated transparent aqueous PAMPSP_4448_ solutions at 5 and 50 °C.

We measured the light scattering intensity (LSI) of the aqueous PAMPSP_4448_ solution with increasing temperature from 5 to 50 °C (Figure 5a). Although %*T* was always constant at 100% regardless of temperature, LSI increased at ≥25 °C. The LSI values for the unsonicated transparent aqueous PAMPSP_4448_ solution at 5 and 50 °C were 0.33 and 7.22 Mcps, respectively. This indicated that dehydration of PAMPSP_4448_ may proceed at ≥25 °C. When the solution was sonicated for 10 min at 13 °C, it did not become turbid. Phase separation occurred when the aqueous solution was sonicated above 25 °C, where the LSI started to increase. The hydrodynamic radius (*R*_h_) distributions for the aqueous PAMPSP_4448_ solutions were measured at 5 and 50 °C, because the temperature dependence on the LSI suggested that the association state changed at temperature above 25 °C (Figure 5b). A bimodal *R*_h_ distribution with unimers (*R*_h_ = 9.5 nm) and aggregates (*R*_h_ = 76.3 nm) was observed at 5 °C. The PAMPSP_4448_ polymer chains were associated with the formation of interpolymer aggregates due to the hydrophobic interactions of the alkyl chains in the counter cation, P_4448_. At 50 °C, a bimodal *R*_h_ distribution was also observed with unimers (*R*_h_ = 7.1 nm) and aggregates (*R*_h_ = 70.4 nm). The aggregate size (*R*_h_ = 70.4 nm) at 50 °C was smaller than that at 5 °C (*R*_h_ = 76.3 nm). As the temperature increased, the polymer chain was dehydrated due to cleavage of the hydrogen bonds between water molecules and the pendant amide or sulfonate groups caused by an increase in molecular motion. Dehydration may proceed preferentially inside the interpolymer aggregates with increasing temperature. The aggregate size decreased at 50 °C due to strong hydrophobic interactions inside the aggregates. As the temperature increased, the *R*_h_ values of PAMPSP_4448_ decreased, while the LSI increased. In general, the LSI depends on the particle number, size, and density in solutions [21,22]. The LSI increased because of the increase in the density of the dehydrated interpolymer aggregates that occurred as temperature increased.

The aqueous PAMPSP_4448_ solution was sonicated at 50 °C to induce turbidity. The solution was then cooled below 5 °C with an ice bath and subsequently heated to 25 °C to prepare the transparent aqueous solution. Furthermore, the cycles of heating to 50 °C, sonication, cooling with an ice bath, and heating to 25 °C were repeated two more times. The *R*_h_ distributions for the transparent aqueous solution at 25 °C after each cycle were bimodal with values of about 7 and 70 nm, which were almost constant regardless of repetition (Figure S6). The aqueous PAMPSP_4448_ solution could be made turbid upon sonication and transparent by cooling three times.

**Figure 6:**
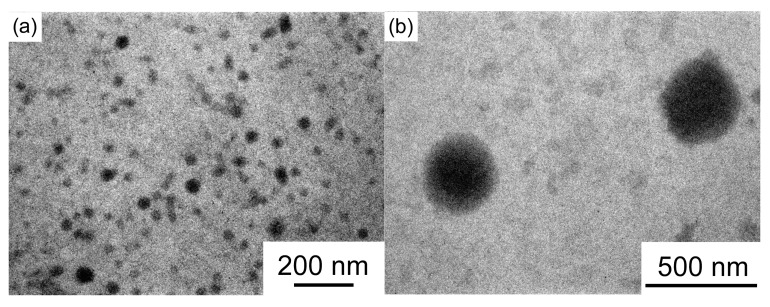
TEM images of aqueous PAMPSP_4448_ solutions under (**a**) transparent and (**b**) turbid conditions.

The aqueous PAMPSP_4448_ solution was sonicated at 50 °C to induce turbidity. A portion of the sample was cooled to a temperature below 5 °C with an ice bath to prepare a transparent solution. TEM samples were prepared using turbid and transparent solutions at 20 °C (Figure 6). Spherical aggregates with an average radius of 38 nm were observed in a TEM image of the transparent solution. The radius was smaller than the *R*_h_ (=ca. 76 nm) of the aggregates estimated from DLS measurements of the transparent aqueous solution at 5 °C. The average radius of the aggregates in the turbid solution was 370 nm, estimated from TEM, which was smaller than that (2.5 μm) estimated from phase-contrast optical microscopy images. The radii estimated from TEM were always smaller than those estimated from DLS and optical microscopy measurements, because the dried samples were observed under reduced pressure for TEM measurements.

### 3.4. Fluorescence Probe

To study microenvironmental polarities inside interpolymer aggregates, we used a hydrophobic pyrene fluorescence probe. The pyrene fluorescence intensity ratio (*I*_3_/*I*_1_) of the 3rd to 1st vibronic peaks increases when the pyrene molecule exists in a hydrophobic environment [23,24]. When pyrene was dissolved in pure water without polymer, *I*_3_/*I*_1_ was 0.55 regardless of sonication and temperature.

**Figure 7:**
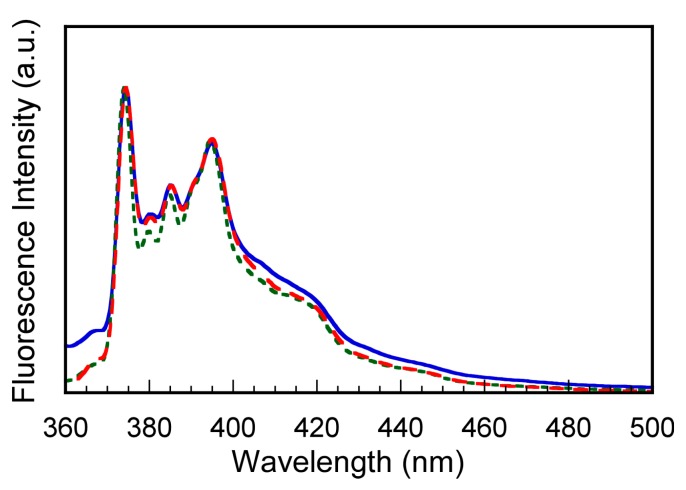
Fluorescence spectra of pyrene excited at 334 nm in the presence of PAMPSP_4448_ in water for transparent aqueous solutions at 5 (---) and 50 °C (---), and a turbid aqueous solution at 50 °C (**—**).

A predetermined amount of PAMPSP_4448_ was dissolved in pyrene saturated pure water. We measured pyrene fluorescence from transparent aqueous solutions at 5 and 50 °C, and from turbid aqueous solution sonicated at 50 °C (Figure 7). The *I*_3_/*I*_1_ ratios for the transparent solutions at 5 and 50 °C were 0.66 and 0.68, respectively. These observations indicated that the pyrene molecules existed in a more hydrophobic environment than in the aqueous phase. The pyrene molecules may be incorporated into hydrophobic domains formed by the association of the alkyl chains in P_4448._ The *I*_3_/*I*_1_ ratio for the transparent solution at 50 °C was slightly higher than that at 5 °C, which suggests that dehydration of interpolymer aggregates proceeded with increasing temperature. This observation agrees with the DLS results (Figure 5), which indicated that dehydration proceeded inside the interpolymer aggregates to decrease the size with increasing temperature. The *I*_3_/*I*_1_ ratio for the turbid solution after sonication was 0.68, which was the same as that for the transparent solution at 50 °C. This indicated that sonication does not change the hydrophobicity inside interpolymer aggregates, while the frequency of collisions increased to form large aggregates.

### 3.5. The Proposed Mechanism of Ultrasound- and Thermo-Responsive Behavior

**Figure 8:**
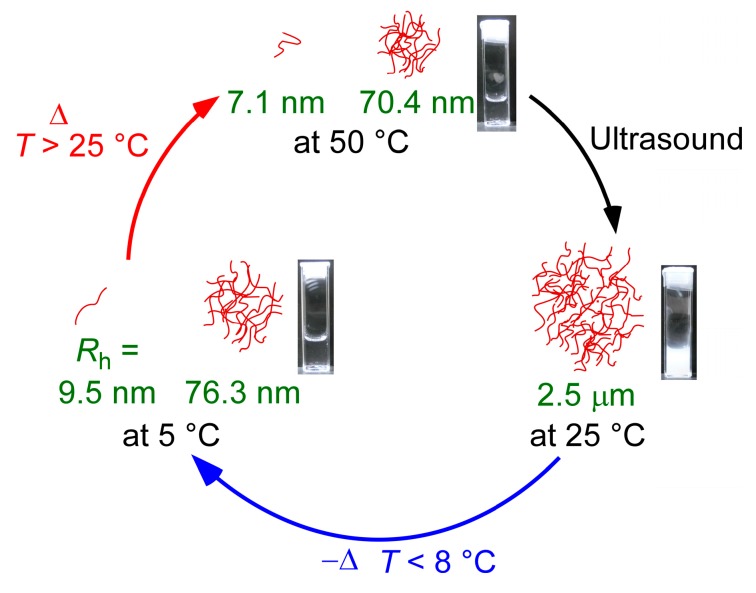
Conceptual illustration of the ultrasound- and thermo-responsive behavior of PAMPSP_4448_ in water.

We discuss the mechanism of the ultrasound- and thermo-responsive behavior of PAMPSP_4448_ in water in conjunction with the above experimental results (Figure 8). Below 25 °C, the pendant amide and sulfonate groups in PAMPSP_4448_ formed hydrogen bonds with water molecules to hydrate the polymer chains. Furthermore, the charged parts of the sulfonate anions and quaternary phosphonium cations were also hydrated. Unimer chains with *R*_h_ = 9.5 nm and interpolymer aggregates with *R*_h_ = 76.3 nm coexisted in water below 25 °C. The aqueous solution where %*T* = 100% at 800 nm was transparent because of the small particle sizes. The interpolymer aggregates were formed by hydrophobic interactions of the P_4448_ alkyl chains. Although a large portion of the hydrophobic alkyl chains in P_4448_ may exist inside the interpolymer aggregates, the pendant hydrated ionic parts and amide groups may be localized on their surfaces. The aggregation number may be constant, and precipitation did not occur due to the hydrated aggregate surfaces. Some hydrated parts of PAMPSP_4448_ may penetrate the hydrophobic domains, because the hydrophilic parts are connected to the main chain.

At ≥25 °C, dehydration progressed due to loss of the hydrogen bonding interactions of water molecules and the pendant amide or sulfonate groups caused by an increase in molecular motion. The hydrophobic interactions inside the interpolymer aggregates were strengthened by dehydration, and the unimer and aggregate sizes decreased to 7.1 and 70.4 nm, respectively. The LSI increased as a result of an increase in the density of the aggregates with dehydration; however, the solution became transparent at 50 °C because of their small particle sizes.

The collision frequency of polymer chains and interpolymer aggregates increased when the somewhat dehydrated polymers were sonicated above 25 °C. When the interpolymer aggregates collide, fusion may occur due to hydrophobic interactions, increasing the size to 2.5 µm. Consequently, the aqueous solution became turbid upon sonication at ≥25 °C. When the turbid solution was cooled below 8 °C, the hydrogen bonds between water molecules and the pendant amide or sulfonate groups in PAMPSP_4448_ reformed. Thus, the polymer chains were hydrated. Consequently, the large aggregates with a radius of 2.5 µm dissociated into unimers and small aggregates with *R*_h_ = 76.3 nm, so that the aqueous polymer solution became transparent. The solution could be repeatedly controlled between turbidity and transparency by sonication and cooling, respectively.

## 4. Conclusions

PAMPSP_4448_ was prepared via RAFT radical polymerization and exchange of counter cations. When the temperature of an aqueous PAMPSP_4448_ solution was increased, it retained its transparency. Interpolymer aggregates were formed from PAMPSP_4448_ via hydrophobic interactions of the pendant alkyl chains in P_4448_ in water. At ≥25 °C, dehydration inside the interpolymer aggregates increased, because hydrogen bonding interactions between water molecules and the pendant amide or sulfonate groups were broken due to increased molecular motion. When the aqueous PAMPSP_4448_ solution was sonicated above 25 °C, the collision frequency of the interpolymer aggregates increased to form large aggregates. Thus, the aqueous solution became turbid upon sonication. When the turbid aqueous solution was cooled below 8 °C, the hydrogen bonds between water molecules and the pendant amide or sulfonate groups reformed to hydrate the polymer chain, and the solution became transparent again. The solution could be repeatedly controlled between turbidity and transparency via sonication and cooling, respectively. No ultrasound- or thermo-responsive behavior was observed for PAMPSP_4444_ with a different alkyl chain length in the pendant counter cation, P_4444_, in water. Therefore, the balance between hydration by hydrogen bonding or electrostatic interactions and hydrophobic interactions is critical for ultrasound- and thermo-responsive behavior.

## Figures and Tables

**Table 1:** Number-average molecular weight (*M*_n_), molecular weight distribution (*M*_w_/*M*_n_), and exchange ratio (ER).

Polymers	*M*_n_ (theory) × 10^4^	*M*_n_ (NMR) × 10^4^	*M*_n_ (GPC) × 10^4^	*M*_w_/*M*_n_	ER *^d^* (%)
PAMPSNa	2.11	2.09	1.85 *^a^*	1.18 *^a^*	-
PAMPSP_4448_	4.73 *^b^*	4.68 *^b^*	0.58 *^c^*	1.17 *^c^*	95.6
PAMPSP_4444_	3.75 *^b^*	3.75 *^b^*	0.36 *^c^*	1.29 *^c^*	99.7

*^a^* Estimated from GPC using a mixed solvent of phosphate buffer at pH 8.0 and CH_3_CN (9/1) as the eluent at 40 °C. *^b^* Calculated at exchange ratio of Na^+^ to P_4448_^+^ or P_4444_^+^. *^c^* Estimated from GPC using 0.1 M LiClO_4_ containing methanol as the eluent at 40 °C. *^d^* Estimated from ^1^H NMR.

## Supplementary Materials

The following are available online at http://www.mdpi.com/2073-4360/10/3/301/s1: Video S1: Ultrasound- and thermo-responsive behavior of PAMPSP_4448_ aqueous solution; Figure S1: Synthesis route of PAMPSP_4448_ and PAMPSP_4444_; Figure S2: ^1^H NMR spectrum for PAMPSP_4444_ in CDCl_3_; Figure S3: Percent transmittance (%*T*) at 800 nm for the aqueous PAMPSP_4448_ solutions at *C*_p_ = 5.0 g/L as a function of the sonication time at 50 °C; Figure S4: Percent transmittance (%*T*) at 800 nm for the aqueous PAMPSP_4448_ solutions as a function of temperature at *C*_p_ = 0.5 (**a**) and 1.0 g/L (**b**). The temperature was increased from 0 to 50 °C (heating; **—**) and decreased from 50 to 0 °C (cooling, ---) at 1 °C/min with stirring. The solutions were sonicated for 10 min at 50 °C and then cooled; Figure S5: Percent transmittance (%*T*) at 800 nm for (**a**) PAMPSP_4444_ in water and (**b**) in 0.1 M aqueous NaCl as a function of temperature at *C*_p_ = 5.0 g/L. The temperature was increased from 0 to 50 °C (—) and decreased from 50 to 0 °C (---) at 1°C/min with stirring; Figure S6: Hydrodynamic radius (*R*_h_) distributions for the transparent aqueous PAMPSP_4448_ solutions at 25 °C. The solution was sonicated at 50 °C. After sonication, the solution was cooled to 5 °C and then heated to 25 °C. *R*_h_ distributions were measured for the transparent aqueous PAMPSP_4448_ solutions at 25 °C after (**a**) one, (**b**) two, and (**c**) three sonication cycles.
