# Supplementary material for: Ultrasound- and Thermo-Responsive Ionic Liquid Polymers
Source: Polymers (Basel). 2018 Mar 11;10(3):301. doi: 10.3390/polym10030301 (PMC6415017; doi:10.3390/polym10030301)
Supplement: Supplementary file 1 [file polymers-10-00301-s001.zip › FinalFile_Ultrasound-ESI.doc]

**Electronic supplementary information (ESI)**

**Ultrasound**- **and Thermo**-**Responsive Ionic Liquid Polymer**

Kohei Itsuki, Yuuki Kawata, Komol Kanta Sharker and Shin-ichi Yusa^*^

Department of Applied Chemistry, Graduate School of Engineering, University of Hyogo, 2167 Shosha, Himeji, Hyogo 671-2280, Japan;

[flowing.yumeniji.quiz@gmail.com](mailto:flowing.yumeniji.quiz@gmail.com) (I.K.); [yuki.chibikitty.1020@gmail.com](mailto:yuki.chibikitty.1020@gmail.com) (K.Y.); [sharkerkomol@diu.edu.bd](mailto:sharkerkomol@diu.edu.bd) (K.K.S.); [yusa@eng.u-hyogo.ac.jp](mailto:yusa@eng.u-hyogo.ac.jp) (S.Y.)

^*^ Correspondence: [yusa@eng.u-hyogo.ac.jp](mailto:yusa@eng.u-hyogo.ac.jp); Tel.: +81-79-267-4954

**Figure S1.** Synthesis route of PAMPSP_4448_ and PAMPSP_4444_.

**Figure S2.** ^1^H NMR spectrum for PAMPSP_4444_ in CDCl_3_.

**Figure S3.** Percent transmittance (%*T*) at 800 nm for the aqueous PAMPSP_4448_ solutions at *C*_p_ = 5.0 g/L as a function of the sonication time at 50 °C.

**Figure S4.** Percent transmittance (%*T*) at 800 nm for the aqueous PAMPSP_4448_ solutions as a function of temperature at *C*_p_ = 0.5 (a) and 1.0 g/L (b). The temperature was increased from 0 to 50 °C (heating; **—**) and decreased from 50 to 0 °C (cooling, ---) at 1°C/min with stirring. The solutions were sonicated for 10 min at 50 °C and then cooled.

**Figure S5.** Percent transmittance (%*T*) at 800 nm for (a) PAMPSP_4444_ in water and (b) in 0.1 M aqueous NaCl as a function of temperature at *C*_p_ = 5.0 g/L. The temperature was increased from 0 to 50 °C (—) and decreased from 50 to 0 °C (---) at 1°C/min with stirring.

**Figure S6.** Hydrodynamic radius (*R*_h_) distributions for the transparent aqueous PAMPSP_4448_ solutions at 25 °C. The solution was sonicated at 50 °C. After sonication, the solution was cooled to 5 °C and then heated to 25 °C. *R*_h_ distributions were measured for the transparent aqueous PAMPSP_4448_ solutions at 25 °C after (a) one, (b) two, and (c) three sonication cycles.
